# Metrics of the normal anterior sclera: imaging with optical coherence tomography

**DOI:** 10.1007/s00417-015-3072-5

**Published:** 2015-06-12

**Authors:** Andreas Ebneter, Nathanael U. Häner, Martin S. Zinkernagel

**Affiliations:** Department of Ophthalmology, Inselspital, Bern University Hospital, and University of Bern, Bern, Switzerland

**Keywords:** Anterior segment OCT, Imaging, Sclera, Age, Intravitreal injection, Glaucoma

## Abstract

**Background:**

To investigate anterior scleral thickness in a cohort of healthy subjects using enhanced depth imaging anterior segment optical coherence tomography.

**Methods:**

Observational case series. The mean scleral thickness in the inferonasal, inferotemporal, superotemporal, and superonasal quadrant was measured 2 mm from the scleral spur on optical coherence tomography in healthy volunteers.

**Results:**

Fifty-three eyes of 53 Caucasian patients (25 male and 28 female) with an average age of 48.6 years (range: 18 to 92 years) were analysed. The mean scleral thickness was 571 μm (SD 84 μm) in the inferonasal quadrant, 511 μm (SD 80 μm) in the inferotemporal quadrant, 475 (SD 81 μm) in the superotemporal, and 463 (SD 64 μm) in the superonasal quadrant. The mean scleral thickness was significantly different between quadrants (*p* < 0.0001, repeated measures one-way ANOVA). The association between average scleral thickness and age was statistically significant (*p* < 0.0001, Pearson *r* = 0.704).

**Conclusions:**

Enhanced depth imaging optical coherence tomography revealed the detailed anatomy of the anterior sclera and enabled non-invasive measurements of scleral thickness in a non-contact approach. The anterior scleral thickness varies significantly between quadrants, resembling the spiral of Tillaux. An association of increasing scleral thickness with age was found.

**Electronic supplementary material:**

The online version of this article (doi:10.1007/s00417-015-3072-5) contains supplementary material, which is available to authorized users.

## Introduction

The sclera is the tension-bearing component of the eye, representing the cornerstone and prerequisite for permanent and stable shape. Despite its bradytrophic nature and low cellular content [1], evidence has emerged in recent years that the sclera may play a major role in ocular disease and pathology [2]. While the focus of investigations has mainly been its posterior part to this point [3–5], the scientific community becomes more attracted to the anterior portion because of the popularity of intravitreal injections, the potential role in alternative transscleral drug delivery [6], new anterior segment operating techniques [7], and the role of the trabecular meshwork in glaucoma [8].

Scleral properties are central in various aspects of glaucoma development and treatment. Biomechanics of the sclera are considered paramount in the pathogenesis of at least some types of glaucomas [9]. Both material properties, such as stiffness, and anatomical features determine the distribution of mechanical stress at the optic nerve head and the lamina cribrosa [10], the putative site of the initial glaucomatous insult [11, 12]. Although not the rate-limiting step, scleral conductivity is a determinant of uveoscleral outflow that is modified by prostaglandin analogues [13]. However, the exact role of the anterior sclera in the pathogenesis of glaucoma is unknown.

Optical coherence tomography (OCT) has become very popular, and has evolved quickly for imaging of the posterior segment. This non-contact method provides high-resolution images of the retina and adjacent structures. The newly available enhanced depth imaging (EDI) modules allow even more in-depth analysis of the choroid and sclera [14]. More recently, OCT has become available for non-invasive assessment of the anterior segment [15]. To date, the focus has primarily been the cornea and anterior chamber structures and dimensions [16]. The aim of the current work was to characterize the sclera adjacent to the limbus using EDI-OCT in healthy eyes.

## Materials and methods

### Subjects

This study was designed as a prospective observational case series. Healthy Caucasian volunteers over the age of 18 years attending the outpatients clinic at a tertiary hospital (Inselspital Bern, Switzerland) were recruited from December 2012 to December 2013. A detailed ocular and general medical history was obtained, and participants underwent a comprehensive slit-lamp examination including intraocular pressure measurement. Individuals with systemic disease potentially affecting the eye (including diabetes mellitus, thyroid disease, and systemic inflammatory conditions) were not eligible. Eyes with previous surgery or signs of relevant ocular pathology such as glaucoma, keratoconus, or high myopia (axial length > 26.5 mm) were excluded.

All procedures performed involving human participants were in accordance with the ethical standards of the institutional research committee and with the 1964 Helsinki Declaration and its later amendments or comparable ethical standards. The Bern University Institutional Review Board and the ethics committee had granted approval (KEK 178/12) for the project. Written informed consent was obtained from all individual participants included in the study.

### Imaging

**Fig. 1:**
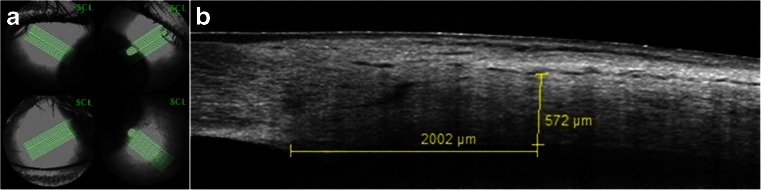
Anterior segment spectral domain optical coherence tomography scans. The photographs illustrate the scan acquisition (**a**) and measurement of the anterior scleral thickness 2 mm posterior to the scleral spur (**b**). In each quadrant, 21 high-resolution B-scans taken approximately perpendicular to the tangent to the limbus were recorded, with the patient holding fixation in the direction opposite to the quadrant recorded (**a**). Scleral thickness was measured between the lamina fusca and the deep episcleral vascular plexus as illustrated in microphotograph **b**

The axial length was measured using the IOL-Master 500 (Carl Zeiss Meditec, Oberkochen, Germany). Spectral-domain optical coherence tomography (SD-OCT) of the perilimbal sclera was obtained with the anterior segment module (Heidelberg Engineering, Dossenheim, Germany) in sclera mode with EDI. In each quadrant, a volume scan consisting of 21 B-scans was recorded comprising the limbus. The orientation of the individual B-scans was approximately perpendicular to the tangent to the limbus (Fig. 1a). The resolution was 768 × 496 pixels (381 KB) with a scan angle of 15°. For image acquisition in each of the four quadrants (inferotemporal, inferonasal, superonasal, superotemporal) patients were asked to maintain gaze position in the opposite direction to the quadrant examined, and care was taken to include the limbus on all scans. The scleral thickness was independently assessed by two masked graders using the Heidelberg eye explorer software (version 1.7.1.0). For each scan area, the scleral thickness was manually measured 2 mm from the scleral spur on the three sections that were of best quality using the caliper tool, and the mean was recorded. The 2-mm distance was chosen in order to compare results to previous work by others [17–19]. The episclera and the conjunctiva were not included in the measurement. The external limit of the sclera can be identified by the deep espiscleral vascular plexus, which manifests as a thin hyporeflective space above the solid scleral tissue (Fig. 1b).

### Statistical analysis

All measurements of continuous variables are reported as means (standard deviation). To ascertain independence of data points, only one eye per patient was included if data from both eyes was available. The eye was randomly selected at the time of statistical data analysis based on a list of integer numbers generated using Microsoft Excel for Mac (Version 14.4.2). Repeated measures one-way ANOVA with Tukey’s correction for multiple comparison post-hoc tests was used to analyze quadrant scleral thickness data. The correlation between scleral thickness and age was analyzed calculating the Pearson *r*. The best-fit regression line was determined using the least-squares method for display in scatter plots. The level of significance was 0.05 (two-sided) for all statistical tests. All analyses were performed using commercial software (Prism 6 for Mac OS X, version 6.0d; GraphPad Software, Inc., San Diego, CA, USA).

## Results

OCTs from 53 eyes of 53 healthy volunteers were included in the analysis. The study population consisted of 25 male and 28 female Caucasian individuals. Participants were on average 48.6 years old (median: 47 years, range: 18 to 92 years). The mean age was 49.4 years (median: 47 years, range: 18 to 90 years) for males, and 47.9 years (median: 47 years, range: 18 to 92 years) for females. The demographic information is shown in Table 1. Axial lengths were evenly distributed with age, and no correlation (*p* = 0.429) was found between these two variables (Supplementary Fig 1).

**Table 1:** Demographic data characterizing patients and eyes included in the study

	Age (SD) [years]	Axial length (SD) [mm]
All (*n* = 53)	48.6 (22.9)	23.97 (1.68)
Male (*n* = 25)	49.4 (22.9)	24.13 (1.27)
Female (*n* = 28)	47.9 (23.3)	23.81 (1.99)
<30 (*n* = 18)	24.8 (3.1)	23.52 (0.91)
30–49 (*n* = 11)	39.0 (7.4)	25.18 (2.16)
50–69 (*n* = 11)	59.5 (6.1)	24.17 (2.22)
> = 70 (*n* = 13)	80.2 (7.5)	23.48 (1.16)

**Fig. 2:**
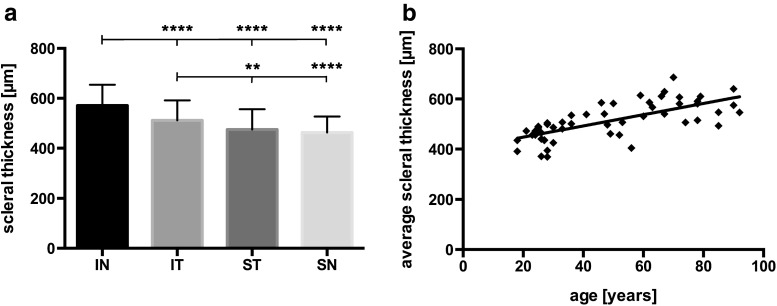
Influence of quadrant and age on scleral thickness. Graph **a** shows the mean anterior scleral thickness for each quadrant [inferonasal (*IN*), inferotemporal (*IT*), superotemporal (*ST*), superonasal (*SN*)]. Repeated measures one-way ANOVA was highly statistically significant (*p* < 0.0001). The anterior scleral thickness in the inferonasal quadrant is significantly thicker than in every other quadrant. The anterior scleral thickness in the inferotemporal quadrant too is significantly different from the superonasal and superotemporal quadrant scleral thickness (post-hoc test with Tukey’s correction for multiple comparison; ** *p* < 0.01, **** *p* < 0.0001). No difference was found between the superonasal and superotemporal quadrants. The scatter plot (**b**) illustrates the positive correlation between scleral thickness and age (*p* < 0.0001, Pearson *r* = 0.704). Average scleral thickness is the mean of all quadrants for each eye. The *line* represents the best-fit linear regression estimate

First, we analyzed scleral thickness and its relationship with individual quadrants. We found that scleral thickness in consecutive quadrants decreased steadily (*p* < 0.0001, repeated measures one-way ANOVA) from the inferonasal to the superonasal quadrant. Post-hoc multiple comparison confirmed that the scleral thickness varied significantly between all but the superotemporal and superonasal quadrants (Fig. 2a). The mean scleral thickness was 571 (84) μm in the inferonasal quadrant, 511 (80) μm in the inferotemporal quadrant, 475 (81) μm in the superotemporal, and 463 (64) μm in the superonasal quadrant.

**Fig. 3:**
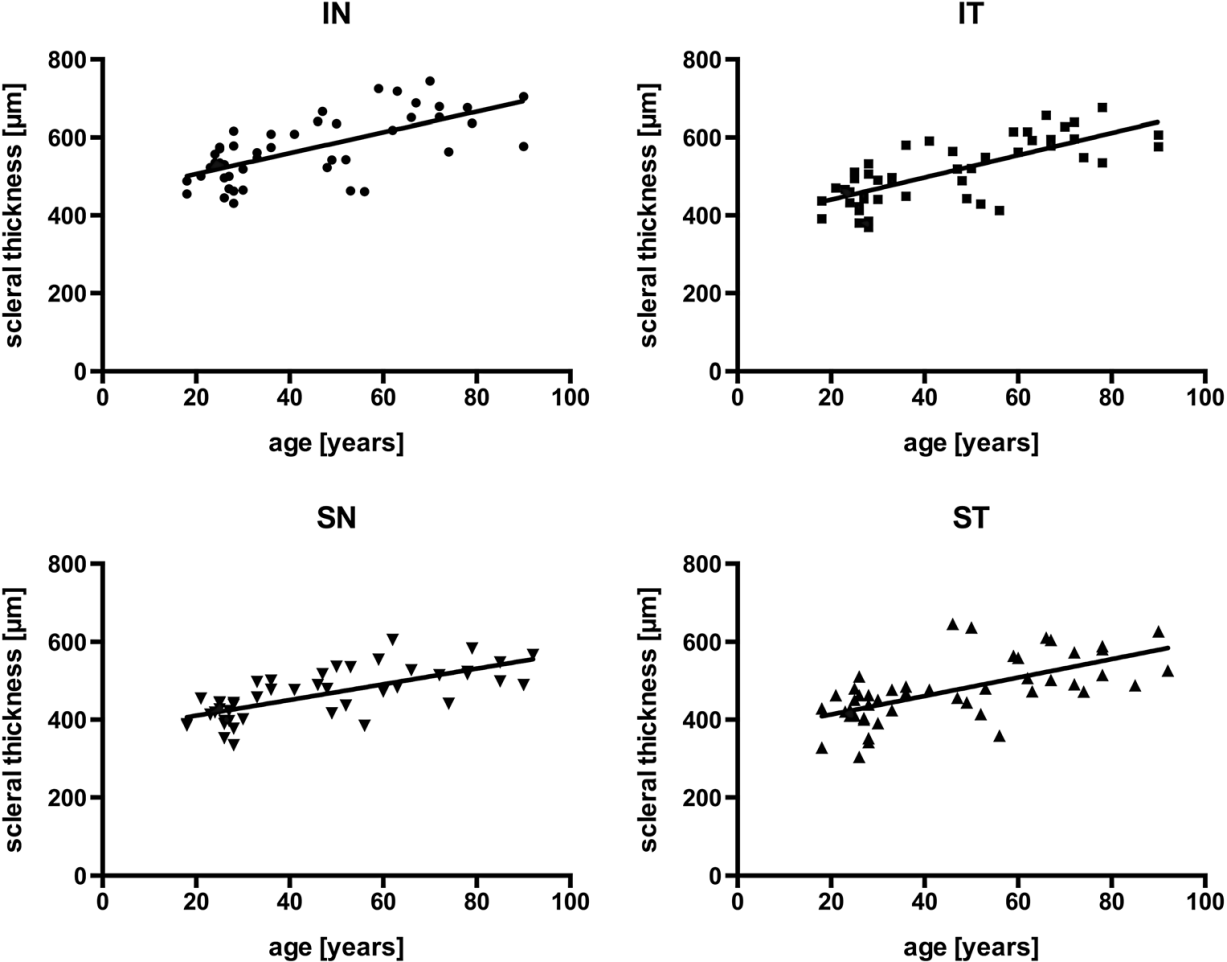
Scatter plots for anterior scleral thickness and age in individual quadrants. The correlation between anterior scleral thickness and age was highly statistically significant in each quadrant [*p* < 0.0001; inferonasal (*IN*), inferotemporal (*IT*), superotemporal (*ST*), superonasal (*SN*)]. *Lines* represent the best-fit linear regression estimates [Pearson *r* = 0.664 (IN), *r* = 0.738 (IT), *r* = 0.703 (SN), *r* = 0.637 (ST)]

Next, we studied the association between age and the average scleral thickness. Interestingly, the correlation was highly significant (*p* < 0.0001, Pearson *r* = 0.704), average scleral thickness being the arithmetical mean of the scleral thickness in each of the four quadrants (Fig. 2b). This correlation was confirmed (*p* < 0.0001 in each quadrant) when data were re-evaluated for each individual quadrant separately (Fig. 3). Data were also analyzed with respect to possible associations between scleral thickness and gender (data not shown). However, no significant relationship was found.

## Discussion

Various techniques have been used in the past to measure scleral thickness [20–24]. Before in-vivo methods became available, investigators examined fixed human donor globes. A fairly consistent picture emerged, and it was confirmed that the sclera is thickest at the posterior pole and the corneoscleral limbus, with its thinnest zone around the equator [21, 22, 25]. The drawbacks of fixed-tissue analysis are susceptibility to post-mortem changes and fixation artifacts. Measurements from fixed tissue were reported to be about 8 % thicker than ultrasound data [22]. Hence, findings might not accurately represent the in-vivo situation. This uncertainty was eliminated when ultrasound biomicroscopy (UBM) and magnetic resonance imaging (MRI) became available. Since the resolution of the latter method was insufficient, UBM turned out to be the preferred choice [20]. However, image acquisition with the UBM technique requires direct contact, and exposure may be challenging because of bulky transducers. The advent of OCT, a non-contact method, has revolutionized posterior segment imaging, not least because of its high resolution, and modifications for anterior segment use have rapidly become available. A plethora of work has been published on applications for cornea, anterior chamber, and trabecular meshwork imaging. Here, we have shown that the anterior segment EDI-OCT also represents a practical and convenient method to assess anterior scleral thickness (AST).

The measurements of the AST obtained in this study corroborate findings from previous work with UBM in vivo and post-mortem studies. It is worthy of note that AST is not uniform around the circumference of the globe but varies between quadrants, being thickest in the inferonasal and thinnest in the superonasal quadrant. This pattern is reminiscent of the spiral of Tillaux, describing the insertion of the rectus muscles with respect to the limbus.

Vurgese et al. [25] conducted a study on human donor eyes fixed in formaldehyde and glutaraldehyde. At the limbus a mean scleral thickness of 500 μm was reported, similar to our results. In a study by Norman et al. [21] on seven enucleated formalin-fixed human globes measured with high-field microMRI, scleral thickness at the corneoscleral limbus was 588 μm. Olsen et al. [22] found a mean scleral thickness at the limbus of 530 μm in 55 formalin-fixed eyes. Lam et al. [20] published data on the AST measured in four meridians 2–3 mm posterior to the scleral spur by UBM in ten healthy eyes from five subjects (mean age 67 years). The mean scleral thickness was 550 μm.

In one of the first UBM studies, Pavlin et al. reported a scleral thickness at the scleral spur of 940 μm in nine normal subjects [23]. In a more recent study, 140 eyes of 140 healthy patients were examined with UBM 2 and 3 mm posterior to the scleral spur in the temporal scleral triangle. The mean AST 2 mm from the limbus was 511 μm [18]. Discrepancies may arise from different definitions of scleral thickness (see below). In a study conducted with the Visante anterior segment OCT, Taban et al. [24] reported a mean scleral thickness of 920 μm in normal eyes 3.5 to 4.0 mm posterior to the scleral spur. They included episclera and conjunctiva in their measurements, which explains some of the difference with this study. Intriguingly, the AST was thickest in the inferonasal quadrant, followed by the inferotemporal, the superotemporal, and then the superonasal quadrant. We have reproduced this finding.

Interestingly, we also found an association between scleral thickness and age in this cohort of healthy Caucasian volunteers. Age-related changes of human sclera material properties such as loss of compliance have been described by several authors [26, 27], and seem to differ between ethnicities [28, 29]. Yet, in previous reports, an association between AST and age was not evident [30, 25]. However, all these data were based on post-mortem studies and analysis was not by quadrants. Thickening of the anterior sclera associated with aging found in this report needs to be confirmed in the future, preferably in a population-based longitudinal approach. Participants in this study were recruited in an outpatient eye clinic setting, and selection bias cannot be ruled out.

Knowing scleral thickness of the anterior segment may be of relevance in a number of clinical scenarios. There is some evidence that repeated intravitreal injections in the same quadrant may cause some degree of scleral thinning [31]. Furthermore, scleral thickness is a major determinant of scleral hydraulic conductivity, which determines resistance to transscleral fluid movements, a component of the uveoscleral outflow. Although not the rate-limiting step of uveoscleral outflow under normal circumstances, transscleral resistance does determine outflow in some pathological states [32]. While the posterior sclera seems pivotal in the pathogenesis of glaucoma [33], the significance of the anterior sclera in this disease is not yet clear. Several studies suggest that central corneal thickness, a parameter predicting progression in ocular hypertensive patients [34], is not related to AST [17, 18, 25], and AST may not be as useful in assessing the glaucoma risk. Even so, Yoo et al. found a correlation between central corneal thickness and AST in patients with normal tension glaucoma, but not in primary open-angle glaucoma subjects or controls [19]. Mohamed-Noorl et al. reported that temporal AST measured by UBM was thicker in ocular hypertensive patients than in subjects suffering from normal tension glaucoma, but not between other subgroups [17]. In this study, however, we only evaluated healthy eyes, and further research will be needed to elucidate the role of the anterior sclera in the pathogenesis of glaucoma.

There are several advantages of OCT in imaging anterior segment structures. Firstly, due to intrinsic properties of light, the resolution is higher for EDI-OCT compared to sound-wave-based methods. Secondly, OCT is a non-contact method and is more practical and convenient than immersion ultrasound. Thirdly, data storage for longitudinal comparison is more straightforward with OCT technology. And lastly, OCT has become more accessible, because it is now routinely used for imaging of the posterior segment. Some devices can be operated in both anterior and posterior segment mode.

The main drawback of EDI-OCT compared to UBM for anterior segment imaging is penetration depth, which precludes examination of the ciliary body. However, some improvement has been achieved with the use of longer wavelengths [35], and most clinically relevant deep anterior segment structures can be visualized on commercial devices [36]. Nevertheless, ultrasound and UBM are still the methods of choice for the assessment of the ciliary body [37]. Tissue differentiation is possible to some degree by standardized echography [38]. The posterior sclera, extraocular muscles, and orbital tissue can be examined with conventional ocular ultrasound. Imaging of the sclera adjacent to the anterior segment with EDI-OCT is feasible to some extent, but can be challenging with the currently marketed OCT devices. Approximately perpendicular exposure to the scan beam is desirable, and the restricted length of the OCT scans may impede simultaneous capture of reference structures, such as the scleral spur. In the current study, specifically, radial scans extending beyond 2 mm posterior to the limbus with the scleral spur as reference structure could not be obtained consistently. However, these constraints might be obsolete with the advent of hand-held OCT devices that become increasingly accessible [39]. It may be relevant to measure scleral thickness in several locations because of topographic variability, not only antero-posteriorly [40, 21, 25], but also circumferentially, as highlighted in this study.

A difficulty of determining scleral thickness in general is the definition and delineation of the sclera on the images. Some authors measure from the retina to the conjunctival surface, whereas others refer to the anatomical definition and exclude uveal tissue, episclera, and conjunctiva. This is partly because it can be ambiguous to determine the interfaces, particularly when using ultrasound-based systems. A common consensus regarding the definition of these structures for imaging would be useful.

In summary, anterior segment OCT is a convenient and non-invasive method to assess the anterior sclera. Scleral thickness varies between quadrants, and there might be a positive association with age. Further prospective studies are warranted.

## Electronic supplementary material

Fig 4 Scatter plot of axial length and age. Axial lengths were evenly distributed with age, and no correlation was found (*p* = 0.429). (GIF 9 kb)

High Resolution (EPS 3612 kb)
