# Supplementary figures and images for: Metrics of the normal anterior sclera: imaging with optical coherence tomography
Source: Graefes Arch Clin Exp Ophthalmol. 2015 Jun 12;253(9):1575–80. doi: 10.1007/s00417-015-3072-5 (PMC4548011; doi:10.1007/s00417-015-3072-5)

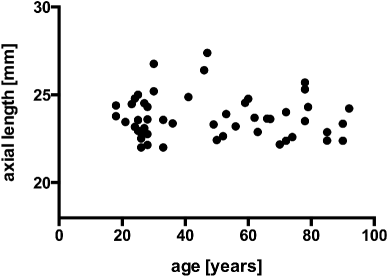

Supplement: Supplementary file 1 — Scatter plot of axial length and age. Axial lengths were evenly distributed with age, and no correlation was found (p = 0.429). (GIF 9 kb) [file 417_2015_3072_Fig4_ESM.gif]
